# The Contagion Effect of Compensation Regulation: Evidence From China

**DOI:** 10.3389/fpsyg.2021.738257

**Published:** 2021-10-01

**Authors:** Jun Shao, Haiyan Zhou, Na Gong, Junzi Zhang

**Affiliations:** ^1^Shanghai Lixin University of Accounting and Finance, Shanghai, China; ^2^The University of Texas Rio Grande Valley, Harlingen, TX, United States; ^3^City University of London, London, United Kingdom

**Keywords:** contagion effect, contagion mechanism, compensation regulation, economic consequence, merging market, compensation

## Abstract

To shed light on whether and how firms changed compensation practices in response to a shift in the environment in which they operated, we examine whether there is contagion effect of executive compensation regulation on state-owned enterprises (SOEs) in the emerging market of China. Specifically, we investigate whether firms not directly affected by the changing regulatory environment nonetheless changed executive compensation in response to the actions of the directly affected firms, which is called contagion effect. We further examine the specific contagion mechanisms and the economic consequences of regulation on compensation. We find that the regulation has a significant effect on compensation gap in central SOEs and a contagion effect on local SOEs but not for non-SOEs. Within SOEs, there is an intra-industry contagion effect of compensation regulation but not an intra-region effect. Further, central SOEs and local SOEs experience reduced firm performance after the compensation regulations, but not the non-SOEs; indicating that the compensation regulation does not have favorable economic consequences for both the directly affected central SOEs and the indirectly affected local SOEs.

## Introduction

The global financial crisis of 2008 triggered a trend in governmental pay regulation world-wide, which led to an increasing role of government regulations in corporate governance and regulations on executive compensations in particular (e.g., Gouldman and Victoravich, 2020). In this study, we investigate whether state-owned enterprises (SOEs)^1^ owned by the local governments in China have mimicked their peers and changed their executive compensation packages subsequent to the pay regulations on SOEs owned by the central-government. As the regulations which cause a spread of regulation shock from central SOEs to local SOEs are originally intended to reduce the pay gap between executives and average employees in the central SOEs, we name such a phenomenon as a *contagion effect.^2^* Compensation regulations in China have existed for some time now. However, the traditional compensation regulations resulted in large compensation gaps, low employee morale, and low productivity. To enhance the alignment between executive compensation and firm performance, the Chinese government in September 2009 mandated that executive salaries in state-owned enterprises (SOEs) owned by the central government should be performance relevant. Nevertheless, how to reduce the compensation gap between executives and average employees is another issue that regulators and policymakers need to resolve. The global financial crisis in 2008 increased the public attention to the compensation gap, which also triggered a series of compensation regulations in China. Among these regulations, the most significant one is issued in February 2013, which regulates executive compensation in central SOEs in all industries. We are interested in whether there is a contagion effect of the 2013 compensation regulation on local SOEs or non-SOEs.

According to Becker and Murphy (2000), the concept of contagion can be traced back to a vast amount of sociology literature on the consistent patterns of group formation. People tend to associate with those who share their language, political views, and demographics. In the corporate world, firms often imitate their peers’ practices, especially in uncertain environments. For instance, companies are more likely to manage earnings after another firm in the same industry or neighborhood announces a restatement (Kedia et al., 2015). There is strong evidence on the usage of relative performance evaluation using peer information matched on both industry and size (Albuquerque, 2009). A number of studies have documented peer group effects on executive compensation (e.g., Bereskin and Cicero, 2013), but few have looked at the contagion effect of compensation regulations. Different from the prior literature which focuses on the direct effectiveness of compensation regulation (Dhole et al., 2015), this study examines whether compensation regulations of central SOEs have a contagion effect on local SOEs. Our study also extends the research on contagion effect of compensation to identify the specific contagion mechanisms. Although the literature (e.g., Bereskin and Cicero, 2013) has documented the role of peer group in executive compensation, few studies have investigated the contagion effect of compensation regulations. Our study documents additional evidence on the contagion mechanisms from the perspective of executive compensation regulations.

Consistent with our hypotheses, we find there is a contagion effect of the 2013 compensation regulation on local SOEs, but not on non-SOEs. We also find that there is an intra-industry, but not intra-region contagion. Both the central SOEs and local SOEs experience reduced firm performance after the compensation regulations, indicating that the compensation regulation does not have favorable economic consequences for either the directly affected central SOEs or the indirectly affected local SOEs.

Our study makes several contributions to the literature on compensation regulation. First, until recently, few studies have examined compensation regulatory policies (except for Dhole et al., 2015). Different from the prior literature which focuses on the *direct* effectiveness of compensation regulation (Dhole et al., 2015), our study examines whether compensation regulations of central SOEs have a *contagion* effect on local SOEs. Second, our study extends the research on contagion effect of compensation to identify the specific contagion mechanisms. Although the literature (e.g., Bereskin and Cicero, 2013) has documented the role of peer group in executive compensation, few studies have investigated the contagion effect of compensation regulations. We find that the contagion effect occurs within the same industry. Our study documents additional evidence on the contagion mechanisms from the perspective of executive compensation regulations. Last but not least, few studies investigate the determinants of executive compensation in Chinese domestic markets (Kato and Long, 2006). Our study extends the existing literature on the determinants of executive compensations by investigating whether government-imposed regulations on central SOEs lead to changes in executive compensation practices in local SOEs.

Our findings have implications for regulators and policymakers. This study confirms government-imposed regulations on central SOEs lead to changes in executive compensation practices in local SOEs. The results show that the compensation regulation has its positive effects, but it also causes the problem of insufficient incentives in medium to long-run for SOE executives, which has a certain downside impact on corporate development. The enforcement of the compensation regulations significantly differs in terms of regional development, income gap, budget deficit, and unemployment rate. The economic consequences of compensation regulation may exceed its original intention. SOE executives are seeking for alternative incentives, such as perks or on-job consumption to make up for insufficient incentives. This study can contributes to the government to make better and more sufficient SOE compensation regulations and to further mobilize the enthusiasm of the executives of SOEs.

The remainder of this study continues as follows. In Section “Executive Compensation Regulations in China,” we introduce the executive compensation regulations. In Section “Literature Review and Hypotheses Development,” we discuss the related literature and develop the hypotheses. Section “Model Specification and Data” presents the data and outlines the empirical approaches employed in the study. In Section “Empirical Tests,” we discuss our primary results, and provide concluding remarks in Section “Conclusion.”

## Executive Compensation Regulations in China

The global economic crisis in 2008–2009, the significant decline in the performance of firms, and the sky-high executive compensation caused widespread discontent among the public. The Chinese government issued series of policies to reduce the compensation gap between executives and employees. In February 2009, the Ministry of Finance issued *The Measures on Executive Compensation of State-Owned and State Holding Enterprises* to regulate the executive pay of central SOEs in the financial and insurance industry. It stipulates that the maximum basic salary of CEOs of financial firms should not be more than five times the average of employees on fixed salaries.

The most prevailing and significant reform incurred in February 2013, when the National Development and Reform Commission, the Ministry of Finance, and the Ministry of Human Resources and Social Security issued *Several Opinions on Further Reform of Income Distribution System* to strengthen the regulation on executive compensation and to enforce regulatory policies on compensation and narrow the compensation gap of SOEs owned by the central government. In particular, it mandates that the growth rate of executive salary should be lower than that of the average employees’ salary. In August 2014, the compensation reform plan for central SOEs was approved, which covered all executives, supervisors, and directors. The reform involves 72 central SOEs. The executive compensation package, which used to include only base salary and performance salary, has now shifted to a structure consisting of a base salary, a performance salary, and an incentive income. The basic salary is based on the average employees’ salary in the previous year, which should be twice as that of the average employees’ salaries. The performance salary is based on the outcome of an annual evaluation, which should be no more than twice the basic salary. The incentive income is based on an assessment of a variety of metrics, which should not exceed 30% of the total annual salary. In this study, we focus only on the contagion effect of the compensation regulation in the second phase because this regulation covers all industries and primarily aims to reduce the compensation gap between executives and employees of central SOEs.

## Literature Review and Hypotheses Development

### The Effect of Regulations on Compensation Gap

Empirical studies find that the compensation structure is asymmetrical, as the marginal increase in executive compensation with improved performance is greater than the marginal decrease with decreasing executive performance. CEOs receive additional bonuses when the firms’ performance increases, but do not pay penalties for declining performance (Jackson et al., 2008). However, few studies have specifically investigated the effect of compensation regulation or its contagion effect.

Most of the listed companies in China were transformed from either central SOEs or local SOEs, where the government dominates the ownership and control. Central SOEs are primarily managed by the Development and Reform Commission of the State Council, while local SOEs are under the charge of local governments. As of July 2017, China has approximately 150,000 SOEs, of which about 50,000 (33%) are owned by the central government and the remainder owned by local governments. The central government directly controls and manages 102 SOEs, of which 66 are listed on domestic and/or international stock exchanges.^3^ These SOEs produce one-fifth of China’s GDP, with their assets accounting for about two-fifths, and their combined profits accounting for three-fifths of all enterprises in China. In the SOEs, executive performance assessment, tenure, and promotion are determined by the government, and the political future of executives is perceived to be more important than other measures. As executive compensation is set by the government, the pay-for-performance sensitivity is weak.

The government has significant and direct influences over the managerial decisions in SOEs, especially with regards to the appointment and termination of top executives. Executives of the central SOEs hold dual roles as a corporate manager and as a government official.^4^ The government plays a decisive role in the development of executive pay packages. When a SOE first submits an application, SASAC reviews it to determine its executive pay. In such a process, executives can use their power in SOEs to seek higher pay (Ke et al., 2012). For central SOEs, the dual role of the executives of SOEs provides themselves the government support, but it also leads to a misalignment of firm performance and executive compensation because central SOEs face relatively great pressure from the public and government regulation (Li and Zhou, 2005), and managers in central SOEs are more likely to be concerned with their political future than the monetary pay.

### Contagion Effect of Compensation Regulation

The term “contagion” refers to imitational behavior whereby peer firms begin redesigning compensation standards after observing a target firm engaging in setting new compensation standards. There are two categories: (a) information transferred through communication channels between the prior adopter and the firm and (b) information inferred from prior adopters belonging to certain reference groups (Reppenhagen, 2010). Research on social influence suggests that a variety of different mechanisms promote the spread of behaviors (e.g., Bikhchandani et al., 1992). Some of these are economically rational, while others are driven by human psychology (Chiu et al., 2013). Early evidence on the behavioral imitation and social learning theory was provided by Bandura et al. (1961, 1963). The social norms explanation for the behavior imitation suggests that when an individual identifies with a social group, the behaviors of others in that social group will have a larger influence on the observer’s social norms. Becker and Murphy (2000) argue that behaviors are most likely subject to social pressures from peers. Such a behavior can also be observed in firms. For instance, managers’ perceptions can be influenced by their superiors’ evaluation styles (Poe et al., 1991). Some researchers propose that executive compensation practices reflect symbolic considerations (Cadman and Carter, 2014), suggesting that firms with relative performance evaluation prefer visible and well-established peers to facilitate the justification of their choices to external constituencies.

According to the Constitution, the central government in China has the power to manage and decide administrative, economic and social fields. Provinces, autonomous regions and provincial-level municipalities are subordinate to the central government. Meanwhile, the central and local governments have implemented the reform of administrative decentralization and fiscal decentralization: local governments have relatively independent economic decision-making power. On the other hand, local governments can share fiscal revenues with the central government. SOEs includes central SOEs and a large number of local SOEs. Central SOEs are generally under the management of the Development and Reform Commission of the State Council and are also under management of local governments (must be at the sub-provincial level or above to be indirectly managed). Since 2003 the State-Owned Assets Supervision and Administration Commission (SASAC) of the State Council has been responsible for managing central SOEs. Each of these provincial-level governments has its own SASAC, which oversees provincial SOEs (local SOEs) (BrØDsgaard et al., 2017). Local SOEs are directly managed by the local government, and the State Council (the central government) generally does not manage local SOEs. The appointment and removal of provincial officials is also determined by the central government.

Within the hierarchy of government, there is a degree of marketization, and local (provincial) governments have a degree of *de facto* discretion (Zhou Y., 2014). Local governments not only need to develop the local economy, but also take on other responsibilities such as local employment, social stability, and public welfare. The significant stake share of local SOEs in most regions provides a platform for local government to participate in economic activities directly. One of the vehicles is that the government could appoint executives with government backgrounds to the local SOEs.

Accordingly, corporate executives of SOEs are required to report to government authorities on a regular basis and are supervised through government audits and reviews. These executives of SOEs are encouraged by the governmental regulatory measures to focus on the overall interests of stakeholders as well as their social functions. Therefore, the executives of SOEs take the role of government officials, who are in the administrative pyramid and thus more concerned with both local revenue and their promotion opportunities (Qian and Roland, 1998). SOEs are mainly to appoint executives, and tournaments are effective institutions as SOE executives’ incentives (Zhou, 2007; Yang et al., 2013). SOE executive is in a position of a relatively closed labor market, and there is a huge gap between inside and outside of SOEs, senior executives are normally not willing to choose to withdraw from existing positions, and promotions are particularly important for SOE’s executives (Zhou, 2004; Huang and Yu, 2009). More important, executives at administrative levels can move horizontally or vertically within the system easily. The pursuit of promotion is mainly to obtain a higher control right, on-job expenditure, and a wider range of resource allocation rights. Therefore, the executives of SOEs take the role of government officials, who are in the administrative pyramid and thus more concerned with both local revenue and their promotion opportunities (Qian and Roland, 1998).

However, local SOEs and central SOEs could be different in terms of practices, regulations, and implementations due to the difference between central government and local governments, if there is any. BrØDsgaard et al. (2017) find that in both SOEs and their publicly listed subsidiaries, administrative experience or political connections appear to increase the likelihood of promotion. However, in the case of central SOE subsidiaries, they find that leaders are more likely to be promoted based on financial performance. Compensation regulation could be an example. The 2013 compensation regulation is issued by the central government on the compensation gap issues in central SOEs, while local SOEs do not have to follow these regulations immediately. While the central government provides a framework of SOE reforms, officials of local governments have to come up with the details of their own plans, which include increasing public listings, improving SOE competitiveness, and monitoring private investment in SOEs in non-regulated industries (Haacke, 2014).

Due to political concerns, executives of local SOEs are most likely to implement compensation regulations even though they are not directly subject to the compensation regulations. The salary regulations could guide executives of local SOEs, which could, in turn, be developed by the local government into detailed rules for a particular region. Although local SOEs can freely choose their peer firms and set up executive salary packages (DiPrete et al., 2010), they have incentives to implement strategic benchmarking behavior in order to avoid the external anger cost and strategically choose central SOEs as their peers and benchmark the latter’s pay structure. Because central SOE heads are “quasi-government officials” rather than professional business executives, compensation regulations are being implemented more strictly in central SOEs. Compared with SOEs, the design of non-SOEs’ executive compensation is more market-orientated. With better performance measures, executives of non-SOEs are relatively highly qualified and thus deserve higher compensation. Since non-SOEs usually operate in highly competitive industries, their survival would depend on the capabilities and efforts of their executives. Therefore, these companies could increasingly rely on their pay systems to attract, retain and motivate executive managers. Compared with local SOEs, the compensation regulation of central SOEs would have less impact on non-SOEs. Hence, we propose Hypothesis 1:

***H1: The contagion effect of compensation regulation of central SOEs is stronger in local SOEs than in non-SOEs.***

### Contagion Mechanisms

Bikhchandani et al. (1992) on social influence suggests that a variety of mechanisms drive the spread of behaviors in a network. Some of these are economically rational, while others are driven by human psychology (Chiu et al., 2013). Rational observers may follow the behavior of others based on direct observation of the action or verbal communication about which action is preferred (Chiu et al., 2013). Companies often imitate the practices of peer firms particularly in an uncertain environment. For example, Kedia et al. (2015) point out that firms are more likely to manage earnings after the public announcement of a restatement by another firm in the same industry or the same neighborhood. There is also strong evidence of on the usage of relative performance evaluation use when peers are matched on both industry and size (Albuquerque, 2009). Observers may follow the behaviors of their peers based on direct observation of the action or verbal communication on which action is preferred. The reason why firms would be influenced by earlier adopters is information-based, which means that prior adoptions provide the information that is relevant to the firm’s decision-making process. The information can be transferred through communication channels between a prior adopter and other firms in the same industry. When faced with uncertainty about an outcome, managers will seek ways to reduce that uncertainty by gathering more information (Olsson, 2010). One source of information is from the observation of other managers who are confronted with similar problems. In performance assessment, managers tend to compare themselves with peers performing similar work (Major and Forcey, 1985). Firms also tend to use benchmarking in CEO compensations (Bizjak et al., 2008; Faulkender and Yang, 2010; Cadman and Carter, 2014).

The other reason why firms would be influenced by prior adopters is spillover-related, which means that prior adoption could change the net benefits of the decision-making of the late adopters. For instance, the prior adoption by an industry rival will increase the likelihood of a firm making a similar decision (Francis and Michas, 2013). Companies in the same industry are natural peers, as they face the same economic and competitive pressures, and perhaps, might be followed by the same financial analysts. Firms in the same industry are also benchmarked against each other by investors and financial analysts. Since companies in the same industry face similar economic pressures and challenges, they are more likely to be interested in similar accounting practices, such as similar mechanisms of earnings management (Becker and Murphy, 2000; Chiu et al., 2013; Kedia et al., 2015). Similar practices could also appear in the executive pay policy. For instance, technology firms typically pay higher compensation to their CEOs in total compensation and stock options than non-technology firms (Dunn et al., 2019).

Firms in the same industry likely face a similar set of intrinsic propensities to adopt; thus, adopting the practice might lead to a similar outcome. The consequences of prior adoptions in the same industry are likely to be especially salient to the firm due to its familiarity with peers in the same industry. Also, the number of adoptions in the same industry is relevant if manager under-weigh their private information signal in the presence of the cumulative influence of other managers’ decisions (Bikhchandani et al., 1992; Reppenhagen, 2010). Therefore, companies in the peer selection process would consider the effect of the industry in performance evaluations (Albuquerque, 2009; Gong et al., 2011). Following the same line of argument, we would expect that the local SOEs in the same industry as central SOEs would be more likely to follow the compensation regulations. Hence, we propose *Hypothesis 2*:

***Hypothesis 2*: *The contagion effect of compensation regulation is stronger for SOEs in the same industry than in other industries.***

Migrants from the same area show that individual decisions can be influenced by the same geopolitical relationship (Kelly and Grada, 2000). Firms located in the same geographical area can also be regarded as peers. Several researchers find that geographical proximity is associated with informational advantages in portfolio decisions (Coval and Moskowitz, 2001) and forecasting accuracy of analysts (Malloy, 2005). Furthermore, companies in the same geographical area make them aware of each other’s business activities. Firms located in the same geographical area belong to diverse industries, face different economic challenges, and may find several practices of their peers to be less relevant to their business. Therefore, contagion in geographical peers is more likely to arise from learning about the costs of making some decisions. The “most common finding in diffusion research is that spatially proximate actors influence each other” (Strang and Soule, 1998, p. 245). Geographical proximity can enable executives to gather more information about another firm’s operations and practices, which can occur due to more exposure to the local press. Hence, we propose *Hypothesis 3.*

***Hypothesis 3: The contagion effect of the compensation regulation is stronger for SOEs in the same region than in other regions.***

## Model Specification and Data

### Model Specifications

Model (1) is used to examine the contagion effect of compensation regulation for central SOEs on local SOEs.


$$\text{GAP}=\alpha +\beta_{1}\,\text{LocalSOE}+\beta_{2}\,\text{LocalSOE}*\text{Reg}+\beta_{3}\,\text{Reg}+\beta_{4}\,\mathrm{Duality}+\beta_{5}\,\text{Stakeholder}+\beta_{6}\,\text{Boardsize}+\beta_{7}\,\text{IndBoard}$$

(1) $$+\beta_{8}\,\text{Growth}+\beta_{9}\,\text{Size}+\beta_{10}\,\text{Lev}+\beta_{11}\,\text{LROA}+\beta_{12}\,\mathrm{Year}\,\mathrm{Dummies}+\beta_{13}\,\mathrm{Industry}\,\mathrm{Dummies}+\beta_{14}\,\mathrm{Firm}\,\mathrm{Fixed}\,\mathrm{Effects}+\varepsilon$$

where the dependent variable GAP^5^ is measured by *GAPD* and *GAPR*. *GAPD* is the logarithm of the difference between average executive compensation and average employee compensation. *GAPR* is the logarithm of the ratio of average executive compensation to average employee compensation. Average executive compensation is defined as the disclosed total compensation of the top three executives divided by three. Average employee compensation is defined as the total employee salary expense (after deducting the top three executive’s salaries) divided by the number of average employees (after deducting the three top executives).^6^ Employee salary expense is calculated as the total employee salaries payable at the end of the period minus the employee salaries payable at the beginning of the period plus cash paid to employees in the current period. *Reg* is a dummy for the post-regulation period, coded as one if the observation is from the post-regulation period and zero otherwise. According to our first hypothesis, we expect β_2_ to be negative.

Consistent with prior studies, we also include control variables such as *Duality, Stakeholder, Boardsize, IndBoard, Size, Growth, LROA*, and dummies for industry sectors. We use the log of total assets (Size) control for firm size because CEOs of larger companies likely receive higher compensation (Dhole et al., 2015). As sales growth is often associated with managers’ pay raises, we include *Growth* and *LROA*, defined as the growth rate of operating income and the previous year’s ROA, respectively. We include *Duality*, *Stakeholder*, *Boardsize*, and *IndBoard* in Model (1) because Sapp (2008) points out that corporate governance features are related to executive compensations. For instance, the literature has documented empirically the effect of corporate governance mechanisms on the relationship between executive compensation and firm performance after the adoption of corporate governance (e.g., Chalevas, 2011). We include industry dummy variables *High-Tech industry*, *Regulated industry*, and *Finance industry* as compensations can vary in different sectors.

Model (2) and Model (3) are used to examine the contagion mechanism of compensation regulation from central SOEs to local SOEs via the same industry and the same region, respectively.


$$\text{GAP}=\alpha +\beta_{1}\,\text{Affected}+\beta_{2}\,\text{Affected}*\text{Reg}+\beta_{3}\,\text{Reg}+\beta_{4}\,\text{Duality}+\beta_{5}\,\text{Stakeholder}+\beta_{6}\,\text{Boardsize}+\beta_{7}\,\text{IndBoard}+\beta_{8}\,\text{Growth}$$

(2) $$+\beta_{9}\,\text{Size}+\beta_{10}\,\text{Lev}+\beta_{11}\,\text{LROA}+\beta_{12}\,\mathrm{Year}\,\mathrm{Dummies}+\beta_{13}\,\mathrm{Industry}\,\mathrm{Dummies}+\beta_{14}\,\mathrm{Firm}\,\mathrm{Fixed}\,\mathrm{Effects}+\varepsilon$$

$$\text{GAP}=\alpha +\beta_{1}\,\text{Province}+\beta_{2}\,\text{Province}*\text{Reg}+\beta_{3}\,\text{Reg}+\beta_{4}\,\text{Duality}+\beta_{5}\,\text{Stakeholder}+\beta_{6}\,\text{Boardsize}+\beta_{7}\,\text{IndBoard}+\beta_{8}\,\text{Growth}$$

(3) $$+\beta_{9}\,\text{Size}+\beta_{10}\,\text{Lev}+\beta_{11}\,\text{LROA}+\beta_{12}\,\mathrm{Year}\,\mathrm{Dummies}+\beta_{13}\,\mathrm{Industry}\,\mathrm{Dummies}+\beta_{14}\,\mathrm{Firm}\,\mathrm{Fixed}\,\mathrm{Effects}+\varepsilon$$

where the dependent variable *GAP* is measured by *GAPD* and *GAPR*, and the test variables are *Affected, Province* and *Reg*. *Affected* is an indicator variable coded as one if there is at least one central SOE in the industry. *Province* is an indicator variable coded as one if there is at least one central SOE in the same province. The definitions of control and other variables are the same as described as earlier. According to Hypothesis 2 and Hypothesis 3, we expect that the coefficient of the interactive terms (*Affected^∗^Reg* and *Province^∗^Reg*), β_2_, is positive. To address possible standard errors cluster issue, we control for firm fixed effect, year fixed effect and industry fixed effect in the regressions.

### Data Collection

Following the requirements of the China Securities Regulatory Commission (CSRC), publicly listed firms started to disclose compensation information on top executives in 2001. Further, starting in 2005, firms were required to disclose separate information on their CEO, board chairman and other executives. Since our focus is on the 2013 compensation regulation, we use the year 2013 as an event year and collect data for the 3 years before and 3 years after 2013. Initially, we select a sample of 91 central SOEs in 2013 and identify 62 local SOEs and 70 non-SOEs using performance score matching approach based *Duality, Stakeholder, Boardsize, IndBoard, Growth, LROA, Size, Lev, High-Tech industry, Regulated industry, and Finance industry*. The sample selection process also includes: (1) removal of observations with missing data; (2) removal of observations of firms whose executives serve less than 1 year; (3) removal of observations in 2013 to facilitate the comparison between the periods before and after the compensation regulation. The final sample consist of 1,208 observations, including 456 observations for central SOEs, 402 observations for local SOEs, and 350 observations for non-SOEs. We obtain executive compensation data from firms’ annual reports and financial and corporate governance data from the WIND and CSMAR databases. To eliminate the effects of extreme values, we winsorize the data at the 1 and 99% for each continuous variable. The sample selection process is summarized in Table 1, Panel A.

**TABLE 1:** Sample selection and descriptive statistics.

Panel A: sample selection	
Central SOEs	91 firms
Non-SOEs identified via Performance Score Matching with Central SOEs	62 firms
Local SOEs identified via Performance Score Matching with Central SOEs	70 firms
Sample firms with data available for analyses	211 firms
Observations in 2010–2016 (excluding 2013)	1208 observations
Including: Central SOEs	456 observations
Local SOEs	402 observations
Non-SOEs	350 observations

**Panel B: Descriptive statistics for pooled sample**						
	**N**	**Minimum**	**Std. dev.**	**Mean**	**Maximum**	**Median**

GAPD	1208	7.283	0.978	12.960	15.169	12.993
GAPR	1208	0.006	0.918	1.997	4.959	1.931
CentralSOE	1208	0	0.437	0.257	1	0
LocalSOE	1208	0	0.492	0.410	1	0
NonSOE	1208	0	0.472	0.334	1	0
Stakeholder	1208	0.000	0.135	0.167	0.562	0.135
Duality	1208	0	0.320	0.116	1	0
Boardsize	1208	1.792	0.176	2.296	2.773	2.303
IndBoard	1208	0.308	0.056	0.376	0.571	0.364
Size	1208	19.084	1.476	22.764	27.248	22.581
LROA	1208	–0.217	0.059	0.038	0.211	0.035
Growth	1208	–0.637	0.753	0.229	5.948	0.101
Lev	1208	0.047	0.206	0.522	1.112	0.536

**Panel C: Descriptive statistics for Sub-sample**						
	**Central SOEs *N* = 456**		**Local SOEs *N* = 402**		**Non-SOEs *N*** = **350**	
	**Mean**	**SD**	**Mean**	**SD**	**Mean**	**SD**

GAPD	12.966	0.845	12.824	1.042	13.110	1.043
GAPR	1.945	0.814	1.913	0.920	2.162	1.019
Stakeholder	0.180	0.137	0.187	0.140	0.126	0.119
Duality	0.086	0.280	0.092	0.289	0.183	0.387
Boardsize	2.319	0.177	2.302	0.176	2.260	0.169
IndBoard	0.374	0.057	0.378	0.059	0.375	0.052
Size	22.905	1.730	22.755	1.248	22.591	1.337
LROA	0.034	0.064	0.036	0.055	0.045	0.058
Growth	0.195	0.697	0.238	0.823	0.264	0.738
Lev	0.547	0.206	0.514	0.201	0.500	0.210

**Panel D: Average Compensation Gaps in Central SOEs, Local SOEs, and Non-SOEs**							
**Variable**	**Period**	**Central SOEs**	**Local SOEs**	**Non-SOEs**	**Central SOEs Vs. Local SOEs (*Z*-test)**	**Central SOEs Vs. Non-SOEs (*Z*-test)**	**Local SOEs Vs. Non-SOEs (*Z*-test)**

GAPD	2010–2012	12.863	12.730	12.815	5.308**	1.317	1.150
	2014–2016	13.068	12.920	13.429	2.236	9.733***	39.159***
	*z*-test	6.885***	8.594***	29.105***			
GAPR	2010–2012	2.020	1.904	2.034	2.614	0.000	1.762
	2014–2016	1.869	1.922	2.299	0.084	17.358***	25.026***
	*z*-test	3.102*	0.139	4.879**			

*, **, *** significant at the 10, 5, and 1% level (2-tailed).

*We apply Wilcoxon rank-sum (Mann-Whitney) test to compare different nature of the enterprise samples.*

*See Appendix 1 for the definitions of the variables.*

### Descriptive Statistics and Correlation Matrix

The descriptive statistics are presented in Panel B of Table 1. The mean (median) of *GAPR* is 1.997 (1.931), indicating that on average the compensation ratio of top executive to the average employee during our sample period is 7.367 (though the median is 6.896). The average (median) of *GAPD* is 12.960 (12.993), indicating that on average the compensation difference between top executives and employees during our sample period is 425,066 (the median is 178,617). About 25.7% observations are on central SOEs and 41% observations on local SOEs; 33.4% observations are from non-SOEs. The sample firms have an average (median) leverage of 52.2% (53.6%). As for the corporate governance features, these firms also have an average (median) logarithm of the number of board members 2.296 (2.303), suggesting that on average there are ten members on board (median is 10). Approximately 37.6% of board members are independent and roughly 11.6% of all observations are from firms with a CEO-chairman duality.

The descriptive statistics for the PSM sub-samples are presented in Panel C of Table 1. For the central SOEs, local SOEs, and non-SOEs sample firms, the mean of *GAPR* is 1.945, 1.913, and 2.162, respectively, indicating that on average the ratio of top executive pay to the average employee during our sample period is 6.994, 6.773, and 8.688 for the central SOEs, local SOEs, and non-SOEs, respectively.^7^ For the central SOEs, local SOEs, and non-SOEs, the average *GAPD* is 12.966, 12.824, and 13.110, respectively, indicating that on average the compensation amount difference between top executives and the average employees during our sample period is RMB 427,624, 371,015, and 493,856, respectively. The central SOEs, local SOEs, and non-SOEs have an average leverage of 54.7, 51.4, and 50%, respectively, and an average operating income growth of 19.5, 23.8, and 26.4%, respectively. The average LROA is 3.4, 3.6, and 4.5% in central SOEs, local SOEs and non-SOEs subsamples, respectively. As for the corporate governance features, the average logarithm of board size is 2.319, 2.302, and 2.260 for these central SOEs, local SOEs, and non-SOE firms, respectively, suggesting that on average there are about ten members on board for all these sample firms. The central SOEs, local SOEs, and non-SOE firms have an average 37.4, 37.8, and 37.5% independent board members, respectively. Non-SOEs sub-sample shows a higher percentage of firms with CEO-chairman duality (18.3%) than the central SOEs (8.6%) and local SOEs (9.2%) subsamples.

Panel D of Table 1 presents the results of a comparative analysis of compensation gaps between central SOEs, local SOEs, and non-SOEs. We use the Wilcoxon rank-sum (Mann-Whitney) test to compare differences in variables among the enterprise samples. During 2010–2012, the mean of *GAPD* (*GAPR*) is 12.863 (2.020) for central SOEs, 12.730 (1.904) for local SOEs, and 12.815 (2.034) for non-SOEs, indicating that before the regulations, the average compensation difference between top executives and employees was 385,771 (7.538), 337,729 (6.713), and 367,691 (7.645) in central SOEs, local SOEs, and non-SOEs. During 2014–2016, the mean of *GAPD* (*GAPR*) was 13.068 (1.869) for central SOEs, 12.920 (1.922) for local SOEs and 13.429 (2.299) for non-SOEs, indicating that after the regulations, the average compensation difference between top executives and employees (the average ratio of top executive pay to the average employee) was 473,544 (6.482), 408,399 (6.835), and 679,424 (9.964). These findings indicate that, in both the pre-adoption and post-adoption periods, the three groups are different from each other in their compensation gap, as measured by *GAPD* and *GAPR*.

Table 2 presents Pearson’s correlations among the variables used in the regression analyses of compensation regulation and compensation gap. The variables *GAPD* and *GAPR* are significantly and positively correlated to each other with a coefficient on 0.356. Central SOE is negatively correlated to *GAPR* with a correlation of –0.059 but not significantly correlated with *GAPD*. Non-SOE is positively correlated with *GAPD* and *GAPR* with a correlation coefficient of 0.053 and 0.101, respectively. All variables have a correlation coefficient of less than 0.400. We also compute variance inflation factors (VIF), all of which are under three. Thus, there are no serious multicollinearity problems in this study.

**TABLE 2:** Pearson correlation coefficients.

	GAPD	GAPR	CentralSOE	LocalSOE	NonSOE	Stakeholder	Duality	Boardsize	IndBoard	Size	LROA	GROWTH	Lev
GAPD	1												
GAPR	0.356	1											
CentralSOE	–0.045	–0.059**	1										
LocalSOE	–0.010	–0.045	–0.490***	1									
NonSOE	0.053*	0.101***	–0.416***	–0.590***	1								
Stakeholder	–0.018	–0.065**	0.1061***	0.031	–0.131***	1							
Duality	0.031	0.128***	–0.059**	0.009	0.046	–0.091***	1						
Boardsize	–0.086***	0.013	0.089***	0.091***	–0.178***	0.053*	–0.133***	1					
IndBoard	0.005	–0.026	0.007	0.004	–0.011	0.038	0.106***	–0.373***	1				
Size	–0.035	0.073**	0.112***	0.033	–0.139***	0.284***	–0.033	0.289***	0.016	1			
LROA	0.037	0.037	–0.040	0.012	0.024	0.037	0.068**	–0.004	–0.023	0.013	1		
Growth	0.119***	0.0377	–0.0803***	–0.010	0.085***	0.026	0.052*	–0.045	–0.003	0.022	–0.026	1	
Lev	–0.041	–0.029	0.081***	0.034	–0.110***	0.029	–0.068**	0.053*	0.006	0.381	–0.151	–0.037	1

*See Appendix 1 for the definitions of the variables.*

*, **, *** indicate, respectively, significant level at the 10, 5, and 1% level.

## Empirical Tests

### Contagion Effect of Compensation Regulation

In this section, we examine whether the regulation on executive compensation in central SOEs is contagious to local SOEs and non-SOEs. Table 3 presents the results of the contagion effect of compensation regulation on local SOEs and non-SOEs. The first two columns show the contagion effect of compensation regulation on local-SOEs. The results show that the coefficient on *Reg* is positive and statistically significant when *GAPD* is used as a proxy for compensation gap, while it is negative and statistically significant when *GAPR* is used, indicating that the compensation regulation did reduce the ratio of executive pay to the average employee but not the dollar amount difference between them. In addition, the coefficient on *LocalSOE^∗^Reg* is statistically insignificant regardless whether compensation gap is measured by *GAPD* or *GAPR*. Thus, there is no difference in the effect of compensation regulation policy between central SOEs and local SOEs, which suggests that while the local SOEs are not the subject of the compensation regulation, the indirect effect they experience is the same as the central SOEs.

**TABLE 3:** The contagion effect of compensation regulations.

	Central vs. Local SOEs		Local Vs. Non-SOEs		Non-SOEs	
Variables	GAPD	GAPR	GAPD	GAPR	GAPD	GAPR
Con	7.57***	–1.87	6.97***	–3.02*	7.50***	–0.12
	(5.41)	(–1.05)	(5.24)	(–1.81)	(4.33)	(–0.05)
Reg	0.28***	–0.36***	0.52***	–0.01	0.27*	0.10
	(3.50)	(–3.56)	(5.59)	(–0.10)	(1.75)	(0.46)
LocalSOE	–0.07	–0.10	0.13*	0.13		
	(–0.98)	(–1.22)	(1.70)	(1.29)		
LocalSOE* Reg	–0.05	0.15	–0.35***	–0.26***		
	(–0.71)	(1.63)	(–4.34)	(–2.56)		
AffectedInd					–0.15	–0.27
					(–0.98)	(–1.22)
AffectedInd*Reg					0.30*	0.62**
					(1.67)	(2.45)
Stakeholder	–0.71**	–0.35	–0.32	0.01	0.35	0.59
	(–2.40)	(–0.93)	(–1.03)	(0.02)	(0.65)	(0.79)
Duality	0.14	0.11	0.05	–0.07	-0.004	–0.24
	(1.51)	(0.88)	(0.61)	(–0.66)	(–0.04)	(–1.54)
Boardsize	0.25	0.37	0.25	0.53	0.17	0.25
	(1.03)	(1.17)	(0.92)	(1.57)	(0.46)	(0.47)
IndBoard	–0.53	0.16	–0.76	0.65	–0.22	0.20
	(–0.93)	(0.23)	(–1.17)	(0.79)	(–0.22)	(0.14)
Size	0.23***	0.14**	0.28***	0.18***	0.28***	0.11
	(4.35)	(2.11)	(5.67)	(2.93)	(4.37)	(1.23)
LROA	0.89***	0.56	1.66***	0.89*	0.07	–0.02
	(2.66)	(1.33)	(4.10)	(1.74)	(0.13)	(–0.02)
Growth	–0.02	0.05	–0.08***	0.004	–0.05	–0.01
	(–0.58)	(1.61)	(–2.90)	(0.12)	(–1.17)	(–0.10)
Lev	–0.67***	–0.66***	–0.91***	–0.62**	–0.88***	–0.26
	(–3.86)	(–3.00)	(–4.57)	(–2.48)	(–3.29)	(–0.66)
Year	Incl.	Incl.	Incl.	Incl.	Incl.	Incl.
Industry	Incl.	Incl.	Incl.	Incl.	Incl.	Incl.
F	6.34	4.45	12.55	5.06	11.64	2.91
R-squared	0.3011	0.1275	0.1940	0.0911	0.1214	0.0009
Samples	858	858	752	752	350	350

*We test the contagion effect from central SOEs to local SOEs and non-SOEs in Table 3, using a sample of 456 observations on central SOEs, 402 observations on local SOEs, and 350 observations on non-SOEs.*

*, **, *** significant at the 10, 5, and 1% level (2-tailed).

*See Appendix 1 for the definitions of the variables.*

The middle two columns show the contagion effect of compensation regulation on local SOEs using a PSM matched sample of both local SOEs and non-SOEs. The results show that the coefficient on *Reg* is positive and statistically significant when *GAPD* is used as a proxy for compensation gap, while it is statistically insignificant when *GAPR* is used. However, the results show that the coefficient on *Local SOE ^∗^ Reg* is negative–the coefficient on *LocalSOE ^∗^ Reg* (β = –0.35, *t* = –4.34, *p* < 0.01) is significantly negative when *GAPD* is used as a measure of compensation gap, and it is also significantly negative (β = –0.26, *t* = –2.56, *p* < 0.01) when *GAPD* is used to measure the compensation gap. Thus, the results suggest that the compensation regulation policy on central SOEs has significantly more contagion effects on the compensation gap in local state-owned enterprises than non-SOEs. Compensation regulation announced by the State Council is enforced more strictly in the central SOEs and it is expected to have an impact on the central SOE executive compensation, which in turn affect the pay gap captured by GAPD. Although local SOEs can freely choose their peer firms and set up executive salary packages (DiPrete et al., 2010), they have political incentives to strategically choose central SOEs as their peers and benchmark the latter’s pay structure. Therefore, it shows negative association of Reg on GAPD. The results are consistent with our expectation that compared with local SOEs, the compensation regulation on central SOEs would have little impact on non-SOEs. The last two columns show whether there is a contagion effect of compensation regulation on non-SOEs. We examine whether the non-SOEs within the same industry as the central SOEs are likely affected by the compensation regulations than other non-SOEs from other industries. The methodology we applied is still DID model. *AffectedInd^∗^Reg* is the interaction term between *AffectedInd* and *Reg*, where *Reg* is a dummy variable which is equal to 1 if the observation is from the post-regulation period, and 0 otherwise. *AffectedInd* is capturing whether there is at least one central enterprise included in the regulation in the industry. The results show that the coefficient on *Reg* is positive and significant when *GAPD* is used as a proxy for compensation gap while it is insignificant when *GAPR* is used, indicating that for non-SOEs the compensation regulation itself does not show any effects to reduce the ratio of executive pay to the average employee or the dollar amount difference between them. Instead, the dollar amount difference between executives and average employees are increased over the sample period. Central SOEs are paying more attention on GAPR. The gap increase in the same industry after regulation for Non-SOEs indicating the regulation has no impact on non-SOEs. Both columns show that the coefficient on *AffectedInd^∗^Reg* is positive and significant when *GAPD* and *GAPR* are used as a proxy for compensation gap, respectively (β = 0.30, *t* = 1.67, *p* < 0.1; β = 0.62, *t* = 2.45, *p* < 0.05, respectively). Thus, we find that the compensation regulation policy on central SOEs has no contagion effects on the compensation gap in non-SOEs in terms of reducing the ratio of executive pay to the average employee or the dollar amount difference between top executives and employees. We do not find any evidence that the compensation regulation affects the gap between executive compensation and employee compensation in non-SOEs within the same industry. However, executives’ compensation in non-SOEs dramatically increased after 2013, therefore, we can see a significantly positive coefficient.

### Contagion Mechanism via Industry and Region

In this section, we examine the contagion mechanism of compensation regulation among SOEs, particularly, whether the contagion effect of compensation regulation is more significant in the same industry or region as central SOEs who are directly subject to the regulations. We test intra-industry contagion mechanism in SOEs in Table 4, using sample of 402 observations on local SOEs and 456 observations on central SOEs. Table 4 shows whether the contagion effect of compensation regulation is more significant in the same industry as central SOEs who are directly subject to the regulations. The results show that the coefficient on *Reg* is positive and significant (β = 0.28, *t* = 3.29, *p* < 0.01) when *GAPD* is used as a proxy for compensation gap while it is negative and significant (β = –0.39, *t* = –3.65, *p* < 0.01) when *GAPR* is used, indicating that the compensation regulation did reduce the ratio of executive pay to the average employee but not the dollar amount difference between executives and average employees, which indicating the regulation has negative association on the pay gap measured by GAPR. This impact is incrementally targeting central SOEs. The results also show that the coefficient on *Affected^∗^Reg* (β = 0.18, *t* = 1.78, *p* < 0.10) is positive and significant when using *GAPR* to measure compensation gap but not significant when using *GAPD*. Relatively speaking, it indeed has impact on local SOEs but the level of pay gap decrease is smaller than central SOEs pay gap decrease. Therefore, it shows a positive coefficient on *Affected^∗^Reg*. The positive sign suggests that the local SOEs have less reductions in the ratio of executive pay to the average employee while compared with central SOEs. The significant sign suggests that the contagion effect of executive pay regulations within the same industry is more obvious when compared with cross-industry. This finding is consistent with the literature, e.g., firms tend to use benchmarking in CEO compensations (Faulkender and Yang, 2010; Cadman and Carter, 2014) by indicating that local SOEs in the same industry as the regulated central SOEs might have incentives to choose central SOEs as their peers (strategically benchmarking).

**TABLE 4:** Intra-industry contagion mechanism in SOEs.

	Central Vs. Local-SOEs	
Variable	GAPD	GAPR
con	7.63***	–1.77
	(5.41)	(–1.00)
Reg	0.28***	–0.39***
	(3.29)	(–3.65)
Affected	0.04	-0.10
	(0.50)	(–1.13)
Affected*Reg	–0.03	0.18*
	(–0.40)	(1.78)
Stakeholder	–0.74**	–0.33
	(–2.49)	(–0.88)
Duality	0.13	0.10
	(1.41)	(0.83)
Boardsize	0.25	0.34
	(1.02)	(1.08)
IndBoard	–0.53	0.12
	(–0.93)	(0.16)
Size	0.23***	0.14**
	(4.27)	(2.09)
LROA	0.87***	0.56
	(2.60)	(1.33)
Growth	–0.01	0.05
	(–0.53)	(1.56)
Lev	–0.68***	–0.65***
	(–3.93)	(–2.97)
Year	Incl.	Incl.
Industry	Incl.	Incl.
F	6.13	4.48
R-squared	0.2910	0.1311
Observations	858	858

*We test intra-industry contagion mechanism in SOEs in Table 4, using sample of 402 observations on local SOEs and 456 observations on central SOEs.*

*, **, *** significant at the 10, 5, and 1% level (2-tailed).

*See Appendix 1 for the definitions of variables.*

Table 5 shows whether the contagion effect of compensation regulation is more significant in the same region as central SOEs who are directly subject to the regulations. When *GAPD* and *GAPR* are used to proxy for the compensation gap, the coefficients on *Province^∗^Reg* are not significant. These results indicate that after the compensation regulations, compared with central SOEs, local SOEs have a similar compensation difference between executives and employees, and a lower ratio of executive pay to the average employee. Moreover, after the compensation regulations, compared with central SOEs, local SOEs have significantly lower compensation growth rate for executives than for employees. These results confirm other researchers’ findings that geographical proximity is associated with informational advantages in portfolio decisions (Coval and Moskowitz, 2001) and forecasting accuracy of analysts (Malloy, 2005). Thus, geographical proximity could enable a local SOE company to learn about another firm’s compensation packages including any adjustments in response to the compensation regulations on central SOEs.

**TABLE 5:** Intra-region contagion mechanism in SOEs.

Variable	GAPD	GAPR
con	7.59***	–1.74
	(5.38)	(–0.98)
Reg	0.28***	–0.38***
	(3.37)	(–3.56)
Province	0.04	–0.08
	(0.60)	(–0.85)
Province*Reg	–0.04	0.16
	(–0.52)	(1.60)
Stakeholder	–0.74**	–0.36
	(–2.48)	(–0.96)
Duality	0.13	0.10
	(1.41)	(0.83)
Boardsize	0.25	0.34
	(1.01)	(1.09)
IndBoard	–0.53	0.11
	(–0.92)	(0.15)
Size	0.23***	0.14**
	(4.28)	(2.06)
LROA	0.87***	0.54
	(2.60)	(1.28)
Growth	–0.01	0.05
	(–0.55)	(1.53)
Lev	–0.69***	–0.64***
	(–3.95)	(–2.94)
Year	Incl.	Incl.
Industry	Incl.	Incl.
F	6.14	4.44
R-squared	0.2931	0.1248
observations	858	858

*We test intra-region contagion mechanism in SOEs in Table 5, using sample of 402 observations on local SOEs and 456 observations on central SOEs.*

*, **, *** significant at the 10, 5, and 1% level (2-tailed).

*See Appendix 1 for the definitions of variables.*

### Additional Test: Compensation Regulation and Firm Performance

In this section, we investigate whether the compensation regulation has economic consequences. The agency-based theory generally supports a positive relationship between executive compensation and firm performance (Nourayi and Mintz, 2008; Ke et al., 2012). The pay-for-performance setting varies with different data, institutions, and model specifications. For instance, Kim and Leung (2007) conclude that compensation incentives cannot resolve agency problems and thus there is a need to have adequate corporate governance mechanisms in place. Different from these prior studies, we investigate whether the government regulation on compensation gap. Following Cunat and Guadalupe (2009), we estimate the effect of the compensation regulation on performance as below:


$$\text{Performance}=\alpha +\beta_{1}\,\text{LocalSOE}+\beta_{2}\,\text{LocalSOE}*\text{Reg}+\beta_{3}\,\text{Reg}+\beta_{4}\,\mathrm{Duality}+\beta_{5}\,\text{Stakeholder}+\beta_{6}\,\text{Boardsize}$$

(4) $$+\beta_{7}\,\text{IndBoard}+\beta_{8}\,\text{LnRev}+\beta_{9}\,\text{Size}+\beta_{10}\,\text{Lev}+\beta_{11}\,\mathrm{Year}\,\mathrm{Dummies}+\beta_{12}\,\mathrm{Year}\,\mathrm{Dummies}+\beta_{13}\,\mathrm{Firm}\,\mathrm{Fixed}\,\mathrm{Effects}+\varepsilon$$

Table 6 shows the results of the compensation regulation on firm performance with *ROE* or *ROA* as a dependent variable for a pooled sample with central SOEs, local SOEs, and non-SOEs. It is divided into four columns for analysis, of which the first column mainly analyses Central SOEs; the second column mainly analyses Local SOEs; the third column mainly analyses Non-SOEs. The fourth column mainly takes Non-SOEs as the control to analyze the influence of SOEs. The results show that the pooled sample firms, after the compensation regulations, experienced a reduced firm performance as indicated by the negative coefficients on *Reg*. When *ROE* is used as a performance measure, the coefficients on *CentralSOE*^∗^*Reg*, *LocalSOE*^∗^Reg, and *NonSOE*^∗^*Reg* are statistically insignificant in all the columns. When *ROA* is used as a performance measure, the same results are presented in all the columns. The results could be caused by the pooled sample, as it includes three different types of firms – central SOEs, local SOEs, and non-SOEs.

**TABLE 6:** Additional test of the effect of compensation regulation on performance.

Variable	ROE				ROA			
Reg	–0.76***	–0.58	–0.13	–0.96***	–0.03***	–0.03***	–0.02***	–0.94***
	(–1.94)	(–1.51)	(–1.11)	(–2.09)	(–3.75)	(–3.65)	(–3.22)	(–3.68)
CentralSOE	0.05			0.15	-0.002			–0.01
	(0.13)			(0.32)	(–0.26)			(–0.64)
LocalSOE		0.13		0.18		-0.004		–0.01
		(0.39)		(0.45)		(–0.53)		(–0.80)
NonSOE			–0.13				0.01	
			(–0.37)				(0.90)	
centralSOE* Reg	0.50			0.73	0.01			0.01
	(1.21)			(1.51)	(0.80)			(1.16)
LocalSOE* Reg		0.08		0.38		0.003		0.01
		(0.22)		(0.84)		(0.43)		(0.95)
NonSOE* Reg			-0.50				–0.01	
			(–1.28)				(–1.19)	
Control variables	Incl.	Incl.	Incl.	Incl.	Incl.	Incl.	Incl.	Incl.
Observations	1208	1208	1208	1208	1208	1208	1208	1208

*We test the compensation regulation on firm performance in Table 6, using a pooled sample of 456 observations on central SOEs, 402 observations on local SOEs, and 350 observations on non-SOEs.*

*, **, *** significant at the 10, 5, and 1% level (2-tailed).

*See Appendix 1 for the definitions of the variables.*

To further investigate whether the results above are caused by the pooled sample, we separately run the regressions for different sub-samples. Table 7 shows the results of the compensation regulation on firm performance with ROE or ROA as a dependent variable for central SOEs, local SOEs and non-SOEs, separately. When ROE is used as a performance measure, the coefficient on *Reg* is –0.51 (*t* = –1.18, *p* > 0.1), –0.40 (*t* = –2.43, *p* < 0.05), –0.33 (*t* = –0.33, *p* > 0.1) for central SOEs, local SOEs, and non-SOEs, respectively. When ROA is used as a performance measure, the coefficient on *Reg* is –0.03 (*t* = –1.84, *p* < 0.1), –0.08 (*t* = –5.58, *p* < 0.01), –0.01 (*t* = –0.66, *p* > 0.1) for central SOEs, local SOEs, and non-SOEs, respectively. It suggests that central SOEs and local SOEs experience reduced firm performance after the compensation regulations but not the non-SOEs, indicating the compensation regulation does not have favorable economic consequences for the directly affected central SOEs and the indirectly affected local SOEs via contagion effects.

**TABLE 7:** Additional test using sub-samples:compensation regulation on performance.

Variable	Central SOEs		Local SOEs		Non-SOEs	
	ROE	ROA	ROE	ROA	ROE	ROA
con	3.56	0.01	–8.68***	–1.49***	–78.87***	–0.01
	(0.44)	(0.03)	(–2.82)	(–5.45)	(–5.03)	(–0.06)
Reg	–0.51	–0.03*	–0.40**	–0.08***	–0.33	–0.01
	(–1.18)	(–1.84)	(–2.43)	(–5.58)	(–0.33)	(–0.66)
Stakeholder	0.40	–0.13*	–0.42	0.03	8.73*	0.08
	(0.19)	(–1.93)	(–0.76)	(0.55)	(1.82)	(1.19)
Duality	–0.12	–0.01	–0.004	–0.01	1.16	–0.02
	(–0.19)	(–0.42)	(–0.02)	(–0.37)	(1.13)	(1.63)
Boardsize	–1.01	0.02	0.71	-0.01	4.86	-0.01
	(–0.66)	(0.33)	(1.32)	(–0.22)	(1.40)	(–0.15)
IndBoard	–0.32	–0.01	–2.67**	–0.02	10.07	0.01
	(–0.09)	(–0.11)	(–2.26)	(–0.22)	(1.06)	(0.07)
Size	–0.10	0.002	0.34***	0.07***	3.11***	0.003
	(–0.31)	(0.18)	(2.89)	(6.47)	(5.38)	(0.30)
LROA	–1.17	0.07	–1.31	0.16**	–8.64*	0.21***
	(–0.62)	(1.22)	(–1.63)	(2.20)	(–1.69)	(2.94)
Growth	0.37**	0.02***	0.08	0.003	0.74**	0.01**
	(2.16)	(3.90)	(1.55)	(0.58)	(2.00)	(2.13)
Lev	1.93*	–0.04	0.001	–0.14***	–11.51***	–0.09**
	(1.90)	(–1.24)	(0.00)	(–3.70)	(–4.49)	(–2.50)
F	1.19	2.02	2.21	6.19	4.52	2.81
R-squared	0.0003	0.0548	0.0027	0.0325	0.0682	0.2000
Observations	456	456	402	402	350	350

*We test the compensation regulation on firm performance in Table 7, using separate sub-samples of 456 observations on central SOEs, 402 observations on local SOEs, and 350 observations on non-SOEs.*

*, **, *** significant at the 10, 5, and 1% level (2-tailed).

*See Appendix 1 for the definitions of the variables.*

### Robustness Test

First, to control for political pressures associated with locations, we add measures on the headquarters geographical locations of SOEs. The government officers in Beijing and major municipal areas could have higher political pressures compared with their counterparts; therefore, we use two alternative measures for the headquarter locations. The first measure is coded as one if the company’s headquarters is located in Beijing and zero otherwise. The second measure is coded as one if the firm’s headquarters is located in Beijing, Shanghai, Guangzhou, or Shenzhen, and zero otherwise. Next, we include the alternative measures in the regression along with its interactive term with *Reg*. The results (not presented in table) show that the coefficients on *Reg* are statistically significant and negative, when we use alternative measures for headquarter location and compensation gap. The result indicates that executives in SOEs headquartered in Beijing and major municipal areas do not show any significant marginal difference regarding the impact of compensation regulation compared with their counterparts.

Second, we use a pooled sample of central SOEs, local SOEs, and non-SOEs and run again the regressions in Table 8. As expected, central SOEs experienced more reductions in the compensation gaps than others after the compensation regulations, as indicated by the significant and negative coefficients on *CentralSOE^∗^Reg*. The only exception is in the first column, where the coefficient on *CentralSOE^∗^Reg* is –0.11 (*t* = –1.61, *p* > 0.1), which is marginally significant when using one tail test. Similarly, local SOEs experienced more reductions in the compensation gaps than others after the compensation regulations, as indicated by the significant and negative coefficients on *LocalSOE^∗^Reg* in most columns. The only exception is in the last third column, where the coefficient on *LocalSOE^∗^Reg* is –0.04 (*t* = –0.53, *p* > 0.1), which is statistically insignificant. In contrast, non-SOEs experienced less reductions in the compensation gaps than others after the compensation regulations. Therefore, the results are qualitatively the same as reported–we find contagion effect on local SOEs, but not on non-SOEs.

**TABLE 8:** Robustness test using a pooled sample.

Variables	GAPD				GAPR			
con	6.97***	6.84***	7.36***	7.24***	–2.15	–2.54*	–1.80	–1.86
	(6.66)	(6.58)	(7.01)	(6.95)	(–1.59)	(–1.88)	(–1.32)	(–1.37)
Reg	0.37***	0.39***	0.24***	0.54***	–0.14	–0.23***	–0.34***	–0.001
	(5.54)	(5.95)	(3.83)	(6.94)	(–1.58)	(–2.73)	(–1.58)	(–0.01)
CentralSOE	0.03			0.09	0.16*			0.24**
	(0.43)			(1.16)	(1.80)			(2.42)
LocalSOE		0.06		0.10		0.03		0.15*
		(0.96)		(1.50)		(0.44)		(1.68)
NonSOE			–0.10				–0.20**	
			(–1.56)				(–2.44)	
CentralSOE* Reg	–0.11			–0.29***	–0.30***			–0.44***
	(–1.61)			(–3.54)	(–3.32)			(–4.15)
LocalSOE* Reg		–0.18***		–0.30***		–0.04		–0.24**
		(–2.85)		(–4.17)		(–0.53)		(–2.54)
NonSOE* Reg			0.30***				0.32***	
			(4.50)				(3.69)	
Stakeholder	–0.49*	–0.48*	–0.43*	–0.43*	–0.23	–0.27	–0.20	–0.19
	(-1.94)	(–1.88)	(–1.72)	(–1.72)	(–0.70)	(–0.81)	(–0.60)	(–0.58)
Duality	0.09	0.10	0.09	0.09	–0.03	–0.02	–0.04	–0.04
	(1.28)	(1.37)	(1.23)	(1.23)	(–0.34)	(–0.24)	(–0.42)	(–0.45)
Boardsize	0.30	0.24	0.24	0.24	0.39	0.35	0.30	0.33
	(1.47)	(1.20)	(1.20)	(1.18)	(1.48)	(1.33)	(1.13)	(1.25)
IndBoard	–0.57	–0.56	–0.58	–0.58	0.12	0.14	0.09	0.09
	(–1.15)	(–1.14)	(–1.19)	(–1.19)	(0.19)	(0.21)	(0.14)	(0.14)
Size	0.27***	0.28***	0.26***	0.26***	0.16***	0.19***	0.17***	0.16***
	(6.80)	(7.20)	(6.65)	(6.63)	(3.26)	(3.76)	(3.28)	(3.07)
LROA	0.72**	0.67**	0.68**	0.68**	0.35	0.30	0.28	0.31
	(2.54)	(2.38)	(2.43)	(2.411)	(0.96)	(0.81)	(0.76)	(0.85)
Growth	–0.03	–0.03	–0.04*	–0.04*	0.03	0.02	0.02	0.02
	(–1.29)	(–1.62)	(–1.65)	(–1.65)	(1.01)	(0.77)	(0.72)	(0.86)
Lev	–0.80***	–0.80***	–0.76***	–0.76***	–0.61***	–0.64***	–0.61***	–0.60***
	(–5.52)	(–5.57)	(–5.32)	(–5.32)	(–3.28)	(–3.42)	(–3.24)	(–3.18)
year	Incl.	Incl.	Incl.	Incl.	Incl.	Incl.	Incl.	Incl.
Industry	Incl.	Incl.	Incl.	Incl.	Incl.	Incl.	Incl.	Incl.
F	14.24	14.63	15.45	14.13	6.06	5.51	6.19	5.85
R-squared	0.2061	0.2054	0.2253	0.2247	0.1030	0.0928	0.0853	0.0933
Observations	1208	1208	1208	1208	1208	1208	1208	1208

*We test the contagion effect of compensation regulation in Table 8, using a pooled sample of 456 observations on central SOEs, 402 observations on local SOEs, and 350 observations on non-SOEs.*

*, **, *** significant at the 10, 5, and 1% level (2-tailed).

*See Appendix 1 for the definitions of the variables.*

## Conclusion

This study provides insight into the contagion effect and economic consequences of compensation regulations in China. Using data on Shanghai and Shenzhen A-share listed companies from 2010 to 2016, we examine whether there is a compensation contagion effect, and if there is a contagion effect, whether the contagion effect is more obvious in the same industry or region. We find that the regulation has a significant effect on the compensation gap in central state-owned enterprises (SOEs). There is a contagion effect on local SOEs, but not on non-SOEs. In SOEs, the contagion effect of compensation regulation works mainly within the same industry. We also find that central SOEs and local SOEs experience reduced firm performance after the compensation regulations, indicating the compensation regulation does not have favorable economic consequences for the directly affected central SOEs and the indirectly affected local SOEs.

One possible limitation of the study is that it didn’t consider the effect of perks or in- on-the-job consumption on executive compensation. The economic consequences of regulations have led managers in SOEs to engage in higher on-the-job consumption. While it is possible that compensation regulations could affect executive compensation, we focus on the indirect contagion effects on local SOEs and non-SOEs. Future studies can explore how individual managers respond to the compensation regulations, whether perks are used in their strategic responses, and whether such practices, if any, would have a contagion effect on other firms.

## Data Availability Statement

The original contributions presented in the study are included in the article/supplementary material, further inquiries can be directed to the corresponding author.

## Author Contributions

JS, HZ, and NG conceived the presented idea and collected available data. JZ verified the analytical methods, developed the theory, and performed the computation. All authors discussed the results and contributed to the final manuscript.

## Conflict of Interest

The authors declare that the research was conducted in the absence of any commercial or financial relationships that could be construed as a potential conflict of interest.

## Publisher’s Note

All claims expressed in this article are solely those of the authors and do not necessarily represent those of their affiliated organizations, or those of the publisher, the editors and the reviewers. Any product that may be evaluated in this article, or claim that may be made by its manufacturer, is not guaranteed or endorsed by the publisher.

## Footnotes

1 Based on the ownership type, Chinese firms can be classified into central SOEs, local SOEs, and non-SOEs. Compared with central SOEs owned by the central government, local SOEs owned by the local governments may enjoy longer grace period and more freedom in complying with reform plans because local governments and local SOEs have more inputs on the details of their own policies.

2 The use of the term contagion here is different from spillover effect studies (Gleason et al., 2008), which focus on investor reactions to other firm behavior and, hence, are about spillover of performance.

3
https://www.export.gov/article?id=China-State-Owned-Enterprises

4 This is different from the situation when the CEO and board chairman are the same person.

5 Hambrick and Siegel (1997) introduce a vertical and horizontal pay dispersion measurements. Vertical pay dispersion captured by the CEO pay divided by the average pay of all level executives. Horizontal pay dispersion is captured by the coefficient of variation of pay for second-level executives. Lin et al. (2003) point that pay gap among executive team refer to the gap between CEO pay and other executive pay, and it measured by the absolute value of pay gap, absolute value of average CEO pay mins average non-CEO pay and absolute value of average CEO pay divided by average non-CEO pay. Kale et al. (2009) summed total, short-term and long-term (including shares) gap compensation to capture pay dispersion and calculate as log (total gap) = log (total compensation of CEO - median value of total compensation of all VPs in the firm-year).

6 According to the 2013 compensation regulation, executives are defined as CEO/President, Vice President, CFO, Chairman of the Board of Directors, and/or other officials defined in the Charters of the company while average employees are defined as employees other than executives mentioned above.

7 Because we use the PSM approach, the subsamples for local SOEs, and non-SOEs we use here are matched samples. Therefore. the descriptive statistics reported here are not for the population of local SOEs and non-SOEs during the same period.

## Appendix 1

Definition of Variables.

**Table:**

Variable	Explanation
GAPD	The measure of compensation gap, calculated as the logarithm of the difference between average executive compensation and average employee compensation.
GAPR	The measure of compensation gap, calculated as the logarithm of the ratio of average executive compensation to average employee compensation.
CentralSOE	A dummy variable which is equal to one if a firm is a central SOE, and zero otherwise
LocalSOE	A dummy variable which is equal to one if a firm is a local SOE, and zero otherwise
NonSOE	A dummy variable which is equal to one if a firm is not SOE, and zero otherwise
Reg	A dummy variable which is equal to one if the observation is from the post-regulation period, and zero otherwise
AffectedInd	An indicator variable which is equal to one if the firm is: (1) a non-SOE, and (2) there is at least one central enterprise included in the regulation in the industry
Affected	A dummy variable that is equal to1 if a firm is in the same industry as the regulated central SOEs and zero otherwise
Duality	A dummy variable that is equal to 1 if the CEO and board chairman are the same person and zero otherwise
Stakeholder	The sum of squares of the share percentages held by the top 3 shareholders
Boardsize	The natural logarithm of total number of directors
IndBoard	The proportion of independent directors on board
Growth	The growth rate of operating income
LROA	ROA in previous year, using Net income divided by total asset in previous year
Lev	The long-term debt divided by total assets
Size	The natural logarithm of total assets
High-Tech industry	A dummy variable that is equal to one if the firm is from a high-tech industry and zero otherwise
Regulated industry	A dummy variable that is equal to one if the firm is from a regulated industry and zero otherwise
Finance industry	A dummy variable that is equal to one if the firm is from a financial industry and zero otherwise
Province	A dummy variable that is equal to one if local SOEs are in the same province as the regulated central SOEs and zero otherwise

*We define high-tech industry based on the *Catalog of High-Tech Industry Statistics* developed by the National Bureau of Statistics of China (2002) http://www.stats.gov.cn/tjsj/.*

*The regulated industries include petroleum, chemical, plastics, metal, non-metallic, electricity, gas, and water production and supply, transportation, warehousing, and information technology industry.*
